# Improving middle school students’ geometry problem solving ability through hands-on experience: An fNIRS study

**DOI:** 10.3389/fpsyg.2023.1126047

**Published:** 2023-03-07

**Authors:** Licheng Shi, Linwei Dong, Weikun Zhao, Dingliang Tan

**Affiliations:** ^1^School of Education Science, Nantong University, Nantong, China; ^2^Jiangsu Institute of Education Sciences, Nanjing, China; ^3^Yulong Road Experimental School, Yancheng, China; ^4^School of Education Science, Nanjing Normal University, Nanjing, China

**Keywords:** problem-solving, geometry learning, hands-on experience, embodied cognition, educational neuroscience, fNIRS

## Abstract

Hands-on learning is proposed as a prerequisite for mathematics learning in kindergarten and primary school. However, it remains unclear that whether hands-on experience aids understanding of geometry knowledge for middle school students. We also know little about the neural basis underlying the value of hands-on experience in math education. In this study, 40 right-handed Chinese students (20 boys and 20 girls) with different academic levels were selected from 126 seventh-grade students in the same school, who learnt “Axisymmetric of an Isosceles Triangle” in different learning style (hands-on operation vs. video observation). Half of them operated the concrete manipulatives while the other half watched the instructional videos. The learning-test paradigm and functional near-infrared spectroscopy (fNIRS) technique were used to compare the differences in geometry reasoning involved in solving well-structured problems and ill-structured problems. Behavioral results showed that hands-on experience promoted students’ performances of geometry problem-solving. Students with lower academic level were more dependent on hands-on experience than those with higher academic level. The fNIRS results showed that meaningful hands-on experience with concrete manipulatives related to learning contents increased reactivation of the somatosensory association cortex during subsequent reasoning, which helped to improve the problem-solving performance. Hands-on experience also reduced students’ cognitive load during the well-structured problem-solving process. These findings contribute to better understand the value of hands-on experience in geometry learning and the implications for future mathematics classroom practices.

## Introduction

Problem solving is a process whereby learners use the knowledge and skills acquired in the past to seek solutions in order to adapt to the needs of the situation (Kahney, 1993). According to Mayer (1998), problem-solving requires students to integrate problem information and maintain mental images of the problem in working memory, and then estimate a reasonable answer and check to make sure everything is accurate. Improving problem-solving performance from the perspective of cognitive psychology has become a research problem that many scholars and mathematics educators pay attention to (Montague et al., 2014). Geometry curriculums in middle school help students develop geometric intuition and promote spatial reasoning ability. Success in geometry problem-solving is highly correlated with students’ math academic achievement in school and beyond (Krawec, 2014).

According to Compulsory Education Mathematics Curriculum Standards (Ministry of Education of the People’s Republic of China, 2012, 2011th Edition) in China, students should have enough time to experience observation, experiment, guess, calculation, reasoning, verification, and other activities in math learning. Mathematical experience needs to be gradually accumulated in the process of doing and thinking. The Compulsory Education Mathematics Curriculum Standards (2022 Edition) point out that it is important to improve teaching methods by mathematical experiments and visualize abstract mathematical knowledge to promote students’ understanding of mathematical concepts and construction of mathematical knowledge. There is accumulating evidence to suggest that hands-on experience plays an important role in math performance. Theories of embodied cognitive hold that cognitive processes are deeply rooted in the body’s interactions with the physical world (Wilson, 2002). According to the theory of embodied mathematics (Giardino, 2018), touching concrete manipulatives contributes to the formation of enriched mental representations, which are beneficial for mathematical learning. In other words, instruction should be more focused on perception that emerge from dynamic interactions, such as folding, drawing, measuring, or manipulating objects. Teachers should encourage students to link these experiences to abstract ideas, rather than concentrate purely on abstract rules (Pouw et al., 2014; Novak and Schwan, 2021). From the viewpoint of perceptual symbol system, the combination of visual, auditory and tactual modalities enriches mental representation (Hutmacher and Kuhbandner, 2018). Memory traces are better understood in terms of sensorimotor encoding (Giardino, 2018; Palmiero et al., 2019), and thus may support the development of more complex understandings (Novack et al., 2014; Kontra et al., 2015; Stull et al., 2018).

Hands-on experience is usually inspired by manipulative materials (Swan and Marshal, 2010; Abrahamson and Sánchez-García, 2016; Nathan and Walkington, 2017). For example, origami/paper folding, the number board game, counters, Cuisenaire rods, Unifix cubes, paper money, pattern blocks, base-10 blocks etc., are often used in the math classroom. A recent study by Freina et al. (2018) found that digital games supported the development and consolidation of visuospatial abilities in students of the last 2 years of the primary school, and such a training would have a positive impact on their mathematics performance. Similar results were obtained by Otten et al. (2020). Fifth-grader students in primary school who worked with the physical balance model more often used advanced algebraic strategies and made a larger improvement in their algebraic reasoning. Hands-on experience blended within instruction was beneficial to understand mathematics concepts and solve real-world problems. Additionally, students who attending the hands-on activities were more likely to recall their experience from a first-person perspective instead of a third-person observer. Shifting between perspectives helped students enrich mental representations and develop a deeper understanding, leading to greater learning gains (Smith, 2018).

However, some researchers dispute the presence of physicality as a requirement and claim that hands-on experience is not always beneficial in mathematics learning (Carbonneau et al., 2013; Rau and Herder, 2021). Thus, despite the ever-increasing researches on embodied learning, there continues to be a need for understanding children’s hands-on experience as it exists in classroom settings.

Firstly, do all students need hands-on experience in mathematical learning? Referring to Boakes (2009) and Burte et al. (2017), students in elementary schools and middle schools took part in hands-on tasks, such as folding and cutting paper, to develop spatial thinking from two-dimensional to three-dimensional. Results showed that all students in elementary schools improved performance on real-world math problems and older students (Grades 5 and 6) improved performance on visual and spatial math problems, while students in middle schools did as well as they did without it. The effectiveness of hands-on activities couldn’t be guaranteed, as age differences should be taken into consideration. According to Piagetian theory, students have the ability to form mental images of abstract concepts and process mental operations of those images to solve mathematical problems given after the age of 11. Some researchers hold the view that hands-on experience wouldn’t enhance math problem-solving at least from the fourth grade onward, for students who have entered the formal operational stage and had the ability of abstract thinking (for an overview see Brown et al., 2009; Carbonneau et al., 2013). However, other researchers provide evidence that hands-on experience brought by physical manipulatives promotes math learning, regardless of the age of the student (Novak and Schwan, 2021). To fill this research gap, the present study is designed to explore whether it is necessary to understand geometric knowledge with the help of hands-on experience for students entering the formal operational stage.

Secondly, does hands-on experience promote or hinder mathematical learning? Researchers hold different views. For mathematics learning that emphasizes abstract thinking training, perceptual richness obtained by manipulatives has been identified as a potential deterrent (McNeil and Uttal, 2009). For example, 11-year-olds and undergraduate students transfer mathematics knowledge more successfully from abstract and symbolic representations than from multiple concrete examples (Kaminski et al., 2009). The perceptual experience that students construct when manipulating objects is often specific to the learning situations, making it difficult to transfer to other contexts or to more abstract knowledge (Brown et al., 2009). However, some researchers pick up the opposite view. As has been discussed above, physical manipulation also can induce psychological simulation of learning materials, which helps to establish a relational link between perceptual experience and abstract symbols, thus promote the development of mathematical thinking with content “visualization” (Novak and Schwan, 2021) and facilitate knowledge retention and transfer (Rau and Herder, 2021). Physicality becomes a cognitive anchor for comprehending abstract knowledge during math learning (Pouw et al., 2014).

Thirdly, how hands-on experience affect geometry problem-solving? Based on the theory of embodied cognition, prior researches concerned about students’ conceptual learning (Pexman, 2017; Rau and Herder, 2021), science learning (Zacharia and Olympiou, 2011), chemistry learning (Stull et al., 2018), physics learning (Kontra et al., 2015), and discussed the mechanism underlying the value of gestures or body movements in problem-solving (Chu and Kita, 2011; Vallotton et al., 2015; Smith, 2018). Few studies have focused on the impact of hands-on experience on student’s geometric reasoning. Existing studies using functional magnetic resonance imaging (fMRI) to examine the state of the cerebral cortex during physical manipulation have found that, compared with observational learning and mental simulation, physical manipulation activates the sensorimotor areas of the frontoparietal cortex to a greater extent, such as primary motor Cortex, premotor cortex, somatosensory cortex, somatosensory association cortex (Bellebaum et al., 2013; Kontra et al., 2015). Whether this is the case for the learning of geometric concepts is unclear. At the same time, meta-analysis confirmed that the neural mechanism of mathematical learning depends on the coordination function of the left prefrontal cortex and parietal cortex area (Artemenko et al., 2019; Molina et al., 2019). If the subject is right-handed, physical manipulation of materials by the right hand and arm consequently results in increased left hemisphere activation (Casasanto, 2009, 2016; Jin et al., 2020). Therefore, in this study, we focused on the frontal and parietal areas of the left hemisphere to explore the neural basis of hand-on experience affects the geometry problem solving.

Functional near-infrared spectroscopy (fNIRS) is a neuroimaging technique that uses near-infrared light to monitor metabolic/hemodynamic changes related to neural activity. Compare to other neuroimaging techniques, fNIRS is portable, lightweight, less sensitive to motion artifacts (Bahnmueller et al., 2014; Quaresima and Ferrari, 2019), which represents a good compromise in terms of spatial and temporal resolution. Moreover, recent development of wireless fNIRS devices has opened the way for new applications in educational neuroscience research, which can be more sensitive and accurate in assessing cognitive function in real-world tasks (Pinti et al., 2015; Soltanlou et al., 2018).

Most of the educational neuroscience research on the effects of hands-on experience use electroencephalography (EEG) and functional magnetic resonance imaging (fMRI). fNIRS imposes fewer physical constraints than EEG and allows free movement in a more natural environment than fMRI, which has great advantages in brain imaging studies that require motor participation in children and is suitable for use in real classroom settings (Piper et al., 2014; Scarapicchia et al., 2017; Soltanlou et al., 2018).

### Research objectives and research questions

In the present study, we aim to investigate whether hands-on experience is a prerequisite for geometry learning in middle school. By hands-on, we talk about the actual and active touch of concrete material related to learning contents under the guidance of math teachers. The opinions on the effect of hands-on experience on children’s math learning are inconsistent, especially for the research focused on students entering the formal operational stage. At the same time, math ability is bound to affect mathematical behavior and cognitive neural processes (Artemenko et al., 2019). But existing studies have different views on the structure of math ability, and viewpoints on the assessment of math ability are also inconsistent. Academic level can reflect math ability to a certain extent (Dupeyrat et al., 2011; Weissgerber et al., 2022), so the present study takes academic level as an important factor when explores the impact of hands-on experience on geometry learning in more detail (Artemenko et al., 2019; Rivella et al., 2021).

In summary, three questions direct this study.

Research Question 1: Does hands-on experience aid middle school students’ understanding of geometry knowledge?

Research Question 2: Is hands-on experience more conducive to geometry problem solving for high-achieving students or low-achieving students?

Research Question 3: What is the neural basis underlying the value of hands-on experience in math education?

## Materials and methods

### Participants

A total of 40 right-handed Chinese students (20 boys and 20 girls, *M*age = 13.58 years, *SD* = 0.32) with different academic levels were recruited from 126 seventh-grade students in the same school in China, who had no prior instruction on related contents. Existing studies have not been consistent in the division of academic levels, such as the top 50% and bottom 50% of students in the class (Chang and Hsin, 2021), or the top 25% and bottom 25% of students (Sermier and Bless, 2013), or the top 20% and bottom 20% of students (Chen et al., 2017) are defined as high and low academic levels, respectively. In order to eliminate the experimental error caused by students with extreme grades, the average of the last two math scores was converted into the standard score in the class. The top 10–27% were defined as high academic level (standard scores = 1.28, *SD* = 0.21), while the bottom 10–27% of the rankings were defined as low academic level (standard scores = −1.24, *SD* = 0.22) in our study. A total of 40 students were selected and arranged into four conditions: A group of 10 students with high academic level under operation condition (operation + H condition), a group of 10 students with low academic level under operation condition (operation + L condition), a group of 10 students with high academic level under observation condition (observation + H condition), and a group of 10 students with low academic level under operation condition (observation + L condition). All students had normal or corrected-to-normal vision and no brain disease. The students and their parents in this study were provided written informed consent before participating in this research and were awarded a present (about $10) for their participation. The present study was carried out in accordance with the ethical standards and requirements established by the local ethical committee of our institute.

### Experimental design

We used a 2 × 2 between-subjects design with the factors hands-on experience (operation/observation) and academic level (high/low). The students of different academic level were randomly assigned to operational condition and observational condition. Dependent variables were the accuracy and reaction time for the problem-solving tasks, as well as the changes of oxyhemoglobin (HbO2) concentration in the left frontal and parietal cortex during the learning process and problem-solving process.

### Experimental tasks

Taking “Axisymmetric of an Isosceles Triangle” as learning content, participants in the operation group turned and folded the paper along the bisector of the top angle to explore the properties of an isosceles triangle, and finally summarized the main points of knowledge in 3-min. Participants in the observation group watched a 3-min video lecture, without operating the concrete manipulatives.

In educational psychology, a problem is a situation in which there is some obstacle between the initial state and the goal state that needs to be overcome. It can be divided into well-structured problem and ill-structured problem (Reed, 2016). Well-structured problems can be represented by a problem space consisting of well-defined initial states and goal states that are connected by legal moves. In contrast, the initial states, the goal states and the intermediate states of ill-structured problems are incompletely specified. In this study, well-structured problems included 10 judgment tasks, of which two were practice tasks and eight were formal tasks. For example, in ΔABC, if AB = BC, AD⊥BC, then ∠BAD = ∠CAD, is it right? Students solved the problems by interpreting and reasoning according to the properties of triangles. Stimuli were randomly presented. Ill-structured problem emphasized the application of knowledge in real life, which tested the participants’ abilities to transfer. The problem was as follows: A worker encountered a problem when building a house. He wanted to know whether the beams of the house were horizontal? An old man solved the problem using an isosceles triangle and a plumb bob tied with a string. The worker didn’t know the reason. How about you?

**FIGURE 1:**
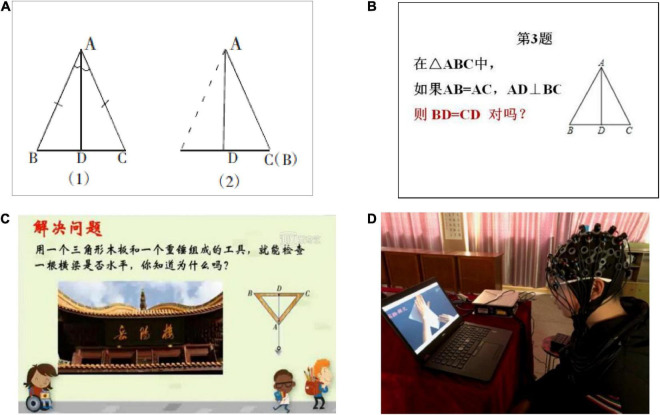
**(A)** Learning content, axisymmetric of an isosceles triangle. **(B)** An example of well-structured problem. **(C)** Ill-structured problem. **(D)** Experimental scenario. See the text for additional details.

The well-structured tasks and ill-structured tasks were developed by math teachers in middle school. The well-structured tasks were selected from after-school practices consistent with the learning content. The ill-structured tasks came from the mathematics textbook of eighth grade. The materials were drawn using Auto-CAD software (see Figure 1).

### Experimental procedure

The participants were tested individually in a dimly lit and noise-free room in the middle school. The lighting is particularly important, given that bright light can affect fNIRS signals (Shadgan et al., 2010). Each participant was seated approximately 70 cm in front of a 21-inch computer monitor (refresh rate 60 Hz, 1,920 × 1,080 resolution) and worn an fNIRS helmet throughout the tasks.

The learning process lasted 3 min. Instructions for the operation group were: “Welcome to our experiment. For the next 3 min, you can fold the isosceles triangle paper beside your hands to explore the nature of an isosceles triangle. If you’re ready, and we’ll start.” The instructions for the observation group were: “Welcome to this activity. For the next 3 min, please watch the video lecture carefully, with no gesture or body movement. If you’re ready, and we’ll start.”

After a 5-min rest, the participants solved the problems. As to the well-structured problem, each trial began with a red fixation cross in the center of the screen for 1 s, followed by the stimulus. When the response was given, the next trial started automatically. There were eight trials in total. As to the ill-structured problem, participants thought for 40 s, then answered verbally. In order to exclude the influence of the experimental sequence, half of the participants solved the well-structured problems first and then the ill-structured problems, while the other half of the participants did the opposite.

**FIGURE 2:**
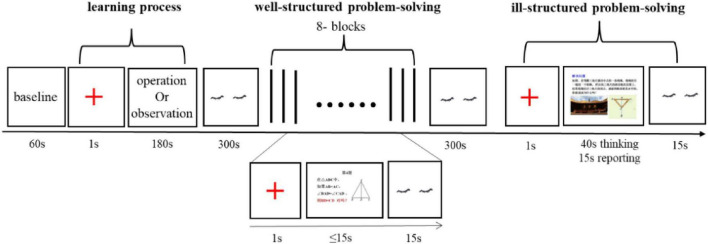
Experimental procedure.

This research lasted about 30 min for each participant. The chart of the experimental procedure was shown in Figure 2.

### Data recording

Behavioral data were captured using the E-Prime 3.0 (Psychology Software Tools).^1^ The software tool recorded information on accuracy and response times in well-structured tasks. The answers of ill-structured tasks were recorded with a voice recorder. Further, three mathematics teachers in middle school were asked to independently rate each subject’s answer with a score ranging from 0 to 10. The average score determined the subject’s final score on the task. The scorer reliability was 0.86.

**FIGURE 3:**
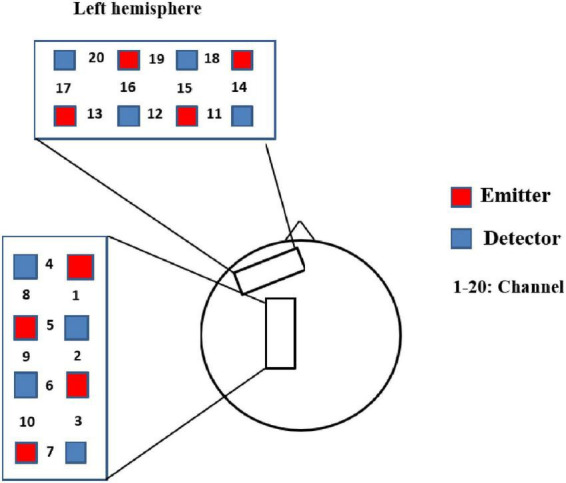
Optical probe positions.

We used LIGHTNIRS system (Shimadzu Corp., Kyoto, Japan) with three wavelengths of near-infrared light (780, 805, and 830 mm) to record the absorption changes of oxyhemoglobin (HbO_2_), deoxyhemoglobin (HbR) and total-hemoglobin (total-Hb). A total of 20 measurement channels (eight sources, eight detectors, source-detector distance was 25.00 mm on average) in a fNIRS helmet covered the prefrontal and parietal cortex in the left hemisphere (see Figure 3 and Table 1).

**TABLE 1:** Positions of the fNIRS channels.

Channels	MNI			AAL		BA	
	x	y	z	Region (L)	Coverage ratio	Region (L)	Coverage ratio
Ch1	−18	−44	78	SPL	0.46	5- SAC	0.77
Ch2	−27	−57	72	SPL	1.00	7- SAC	0.94
Ch3	−24	−66	68	SPL	0.97	7- SAC	1.00
Ch4	−24	−26	75	PCG	0.60	4- PMC	0.78
Ch5	−33	−45	72	SPL	0.69	5- SAC	0.59
Ch6	−34	−57	69	SPL	0.94	7- SAC	0.85
Ch7	−29	−68	64	SPL	1.00	7- SAC	1.00
Ch8	−40	−28	70	POCG	0.68	4- PMC	0.62
Ch9	−42	−44	66	IPL	0.42	40- Wernicke’s area	0.71
Ch10	−40	−58	63	SPL	0.59	7- SAC	0.61
Ch11	−22	53	41	SFG	0.83	9- DLPFC	0.87
Ch12	−45	36	37	MFG	0.94	9- DLPFC	0.59
Ch13	−56	11	37	MFG	0.72	9- DLPFC	0.66
Ch14	−10	66	28	SFG	0.57	10- FPC	0.92
Ch15	−38	56	24	MFG	0.96	10- FPC	0.90
Ch16	−53	36	21	IFGtriang	0.83	46- DLPFC	0.92
Ch17	−63	6	28	MFG	0.47	44- Broca’s area	0.86
Ch18	−26	68	10	SFG	0.86	10- FPC	1.00
Ch19	−46	53	5	MFG	0.53	10- FPC	0.65
Ch20	−57	31	9	IFGtriang	1.00	45- Broca’s area	0.64

The location data listed in this table is the area with the greatest coverage probability. SPL, superior parietal lobe; IPL, inferior parietal lobe; POCG, postcentral gyrus; SFG, superior frontal gyrus; MFG, middle frontal gyrus; IFGtriang, Inferior frontal gyrus, triangular part; DLPFC, dorsolateral prefrontal cortex; SAC, somatosensory association cortex; PMC, primary motor cortex; FPC, frontopolar cortex.

In order to determine the anatomical areas underneath the channels, the 3D localizer (FASTRAK, Polhemus, Colchester, VT, USA) were utilized to confirm the positions of CZ, NZ, Al, AR, and the probes. The coordinate of the channels was positioned and aligned with the automatic anatomical labeling (AAL) atlas and Brodmann’s area (BA) in SPM software. See Table 1 for details.

### Data analysis

Two-way analysis of variance (ANOVA) was conducted on the accuracy (ACC) and response time (RT), to determine whether there were significant differences between the four different conditions in well-structed problem solving tasks. As to ill-structed problem, ACC was analyzed.

Functional near-infrared spectroscopy data analysis was carried out using NIRS_SPM software (Ye et al., 2009) and matlab2014a (MathWorks, Natick, Ma, USA). Modified Beer-Lambert law was used to transform measured optical densities into hemoglobin concentration. For each channel, the original fNIRS data were low-pass filtered and high-pass filtered through wavelet-based methods of hemodynamic response function (HRF) and Discrete Cosine Transform (DCT) with a cutoff period of 128 s to remove motion artifacts and physiological noise induced by heartbeat, breathing cycle and low frequency oscillations of blood pressure (Ye et al., 2009; Tak and Ye, 2014). The mean changes in HbO_2_ and HbR concentration were obtained using the last 30 s of the resting state before the beginning of the task as a baseline. Finally, the general linear model (GLM) was used to calculate the individual β values for each channel, participant, and task. We focused on HbO_2_, which has the highest sensitivity to changes in cerebral blood flow (Okamoto et al., 2004; Hoge et al., 2005), to assess the participants’ brain activation. All the results were corrected using the false discovery rate (FDR), and the adjusted significant level of *p*-value was set at 0.05 (Singh and Dan, 2006). With the help of EasyTopo toolbox (Tian et al., 2013), two-dimensional plane images were output to display the location of activated brain regions.

## Results

Considering that behavior results need to correspondence to fNIRS data, two participants had to be excluded because they didn’t follow the instructions properly or because of technical problems in response recording. Math standard scores were analyzed by 2 × 2 ANOVA. There were significant differences between academic level [*F*_(1,34)_ = 613.00, *p* < 0.001, η^2^_p_ = 0.95] but not hands-on experience [*F*_(1,34)_ = 0.01, *p* > 0.05], and no significant interaction effect was found [*F*_(1,34)_ = 0.13, *p* > 0.05]. It meant that the manipulation of variables was still valid.

### Behavioral results

#### Well-structured problem-solving

A two-way ANOVA with hands-on experience and academic level as between-subjects factors, showed a significant main effect of the hands-on experience on the accuracy [*F*_(1,34)_ = 4.34, *p* = 0.045, η^2^_p_ = 0.11, 95% CI: 0.53∼0.73] and reaction time [*F*_(1,34)_ = 4.39, *p* = 0.044, η^2^_p_ = 0.11, 95% CI: 6847.76∼8273.06]. The main effect of academic level was also significant on the accuracy [*F*_(1,34)_ = 6.04, *p* = 0.019, η^2^_p_ = 0.15, 95% CI: 0.53∼0.74] and reaction time [*F*_(1,34)_ = 9.85, *p* = 0.004, η^2^_p_ = 0.23, 95% CI: 6687.32∼8411.80]. Interaction effect between hands-on experience and academic level was not found, suggesting overall a consistent trend in learning gains between the conditions.

#### Ill-structured problem-solving

We only recorded the accuracy of ill-structured problem-solving, and analysis of variance showed that both hands-on experience [*F*_(1,34)_ = 22.31, *p* < 0.001, η^2^_p_ = 0.39, 95% CI: 3.98∼6.40] and academic level [*F*_(1,34)_ = 90.90, *p* < 0.001, η^2^_p_ = 0.73, 95% CI: 3.22∼7.18] had significant main effects. There was a significant interaction effect between hands-on experience and academic level [*F*_(1,34)_ = 5.37, *p* = 0.027, η^2^_p_ = 0.14, 95% CI: 4.17∼7.73]. Further simple effect analysis found that, for those with low academic level, the accuracy of the operation group was higher than that of the observation group [*F*_(1,34)_ = 20.79, *p* < 0.000, η^2^_p_ = 0.31], with no significant difference in high academic level students [*F*_(1,34)_ = 2.89, *p* = 0.098]. The accuracy of high-achieving students was higher than low-achieving students, both in the operation group [*F*_(1,34)_ = 27.48, *p* < 0.000, η^2^_p_ = 0.45] and in the observation group [*F*_(1,34)_ = 66.73, *p* < 0.000, η^2^_p_ = 0.66]. Descriptive data are shown in Table 2.

**TABLE 2:** Behavioral performance of problem-solving tasks.

Measures (*n* = 38)	Well-structured problem-solving		Ill-structured problem-solving
	Accuracy	Reaction time (ms)	Accuracy
Operation + H condition (*n* = 10)	0.72 (0.11)	6882.79 (844.43)	7.10 (0.74)
Operation + L condition (*n* = 10)	0.63 (0.15)	7615.17 (858.01)	4.80 (1.23)
Observation + H condition (*n* = 9)	0.65 (0.10)	7316.28 (957.40)	6.33 (0.71)
Observation + L condition (*n* = 9)	0.54 (0.11)	8383.99 (875.25)	2.56 (1.13)

H, high academic level; L, low academic level.

### Functional near-infrared spectroscopy results

**FIGURE 4:**
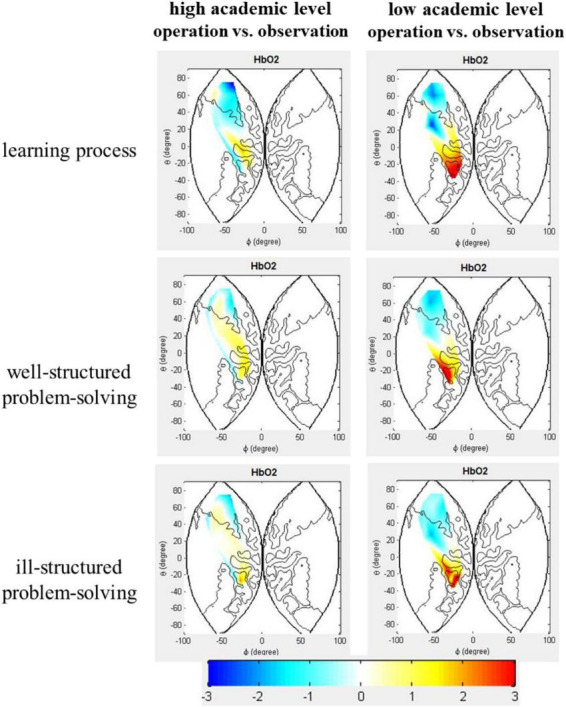
Brain activation images during the learning process and problem-solving process.

We analyzed the HbO_2_ data in all 20 channels measured by fNIRS (see Figure 4). During the learning process, the result demonstrated that the main effects of hands-on experience were significant in SAC [Ch5: *F*_(1,34)_ = 9.94, *p* = 0.030, η^2^_p_ = 0.23, 95% CI:−0.002∼0.012] and PMC [Ch8: *F*_(1,34)_ = 14.02, *p* = 0.020, η^2^_p_ = 0.29, 95% CI:−0.002∼0.014]. A significantly greater activation was founded in the operation group than in the observation group. The main effect of academic level was significant in DLPFC [Ch13: *F*_(1,34)_ = 12.75, *p* = 0.020, η^2^_p_ = 0.27, 95% CI:0.004∼0.013]. HbO_2_ variations in the low-achieving group were significantly higher than that in the high-achieving group. Interaction effect between hands-on experience and academic level was significant in SAC [Ch10: *F*_(1,34)_ = 10.83, *p* = 0.040, η^2^_p_ = 0.24, 95% CI:−0.002∼0.011]. Further simple effect analysis illustrated that for those with low academic level, a significant increase of HbO_2_ concentration was observed in Ch10 [*F*_(1,34)_ = 15.43, *p* < 0.000, η^2^_p_ = 0.31] in the operation group compared to the observation group. No significant difference between the learning conditions was found in high-achieving students. Meanwhile, the HbO_2_ variations in the high-achieving group were significantly higher than that in the low-achieving group in Ch10 [*F*_(1,34)_ = 13.05, *p* = 0.001, η^2^_p_ = 0.28] in the observation group, with no significant difference in the operation group.

During the problem-solving process, for the left SAC, the results demonstrated that the main effects of the hands-on experience were significant both in the well-structured problem-solving tasks [Ch5: *F*_(1,34)_ = 9.80, *p* = 0.04, η^2^_p_ = 0.22, 95% CI:0.002∼0.010; Ch6: *F*_(1,34)_ = 11.57, *p* = 0.04, η^2^_p_ = 0.25, 95% CI:0.001∼0.010] and in the ill-structured problem-solving tasks [Ch6: *F*_(1,34)_ = 12.22, *p* = 0.020, η^2^_p_ = 0.26, 95% CI:0.002∼0.010; Ch7: *F*_(1,34)_ = 12.21, *p* = 0.010, η^2^_p_ = 0.15, 95% CI: 0.002∼0.010]. A significantly higher activation in the operation group than in the observation group. No other significant effects were discovered (*ps* > 0.05). For the left DLPFC, the main effects of hands-on experience and academic level were significant in the well-structured problem-solving tasks (*ps* < 0.05). HbO_2_ variations in the low-achieving group were significantly higher than that in the high-achieving group [Ch13: *F*_(1,34)_ = 12.58, *p* = 0.02, η^2^_p_ = 0.27, 95% CI:0.003∼0.008]. HbO_2_ variations in the observation group were significantly higher than that in the operation group [Ch11: *F*_(1,34)_ = 10.36, *p* = 0.02, η^2^_p_ = 0.23, 95% CI:0.001∼0.011].

### The relationship between brain activity and behavioral performance

Based on the fNIRS results, we concerned the brain regions of interest (ROI) correspond to the following Brodmann divisions: left SAC(Ch1,2,3,5,6,7,10), left DLPFC(Ch11,12,13,16), and HbO_2_ concentration is averaged between channels in each ROI before statistical analysis.

Pearson’s correlation analyses revealed a significantly correlation between the left SAC activity and problem-solving performance. The activation in left SAC was positively correlated with the accuracy (*r* = 0.448, *p* = 0.005) and negatively correlated with the reaction time (*r* = −0.441, *p* = 0.006) of well-structured problem-solving. Moreover, the activation of left SAC was positively correlated with the quiz score of ill-structured problem-solving (*r* = 0.470, *p* = 0.003). Obtaining this result proved that the extent of the sensorimotor brain system’s involvement caused by hands-on experience is related to geometry reasoning.

Pearson’s correlation analyses also revealed that the activation in left DLPFC was positively correlated with the reaction time of well-structured problem-solving (*r* = 0.432, *p* = 0.007), and negatively correlated with the quiz scores of ill-structured problem-solving (*r* = −0.471, *p* = 0.003).

## Discussion

In the present study, we demonstrated positive effects of hands-on experience on geometry knowledge acquisition in middle-school. The knowledge of properties of the isosceles triangle was implemented in two different ways (hands-on operation vs. video observation). Students’ geometry reasoning was examined through solving well-structured problem and ill-structured problem.

Our results showed that students who gained the knowledge of “properties of isosceles triangle” by hands-on operation outperformed students who observed the same phenomena, both in well-structured problem-solving tasks and in ill-structured problem-solving tasks, indicating the beneficial role of hands-on experience in geometry learning. More importantly, hands-on experience improved the students’ performance across ability levels. Therefore, it could be reasonably against the viewpoints that both perceptual and interactive richness of instructional manipulatives would hinder symbolic inferences and compromise application of knowledge to new situations, thus resulting in lower learning outcomes (Brown et al., 2009; Kaminski et al., 2009; Pouw et al., 2014). These results supported the findings of Kontra et al. (2015) in physics learning, Stull et al. (2018) in chemistry learning, and Zacharia and Olympiou (2011) in science learning. Hands-on experience tied to the to-be-learned content could promote learning, even if learners have already had the ability to think abstractly.

However, our main interest was how hands-on experience enhance student’s mathematics learning. Consistent with many previous studies, the operation group explored the characteristics of the isosceles triangle by folding and turning the paper, thus increasing the activation of the primary motor cortex (Bellebaum et al., 2013; Kontra et al., 2015). Compared with observational learning, hands-on operation required the integration of vision, haptics and other sensory information, and made the somatosensory association cortex activated. Later on, SAC reactivated during well- structured and ill-structured problems solving, contending that memory traces capture and reflect the perceptual components of past experience. Such evidences supported the reactivation hypothesis (Nyberg et al., 2001; Ianì, 2019), which amounted to the idea that memory trace consisted in a reactivation of the same sensorimotor regions initially activated in its perception. Similar results were obtained by Kontra et al. (2015). Activation in SAC was also positively correlated with performances in problem solving. Our findings offered a possible explanation for how hands-on experience enhanced understanding of geometry knowledge. Hands-on operation (relative to observation) increased activation of sensorimotor systems important for representing and processing geometry terms. This activation, in turn, enhanced understanding of the torque and isosceles triangle (as assessed *via* our quiz).

Math ability affects mathematical problem-solving (Krawec, 2014; Artemenko et al., 2019). This study further obtained consistent results that students with high academic level outperformed the students with low academic level in problem-solving tasks. In addition, recent studies have consistently suggested that activation in left DLPFC scales linearly with mental workload (Ayaz et al., 2012; Fishburn et al., 2014), and DLPFC activity also increased during mental effort anticipation of hard tasks as compared to easy tasks (Vassena et al., 2019). In this study, left DLPFC activated significantly higher in the low-achieving students than in the high-achieving students during the learning process and well-structed problem-solving process, no matter in which learning style (both hands-on operation and video observation), indicating that low-achieving students got more mental workload in geometry learning. Left DLPFC activated significantly higher in the observation group than in the operation group in the well-structed problem-solving tasks, which implied that hands-on operation served to off-load the demand of reasoning in the mind on to external objects (Stull et al., 2018), and thus supported the development of more complex understandings and improved geometry problem-solving. Recent psychophysical studies have provided evidence that each modality (visual, auditory, tactual) has its own working memory. If multiple modalities are employed compared to the use of a single modality for a same amount of information, the cognitive capacity increases and thus the cognitive load is reduced (Zacharia et al., 2012).

Hands-on experience can reduce the difference of problem-solving ability formed by academic level. When solving the ill structured problems that come from real life situations and require the participation of advanced cognitive processes, students with low academic level in the operation group outperformed those in the observation group. However, for those with high academic level, there was no significant difference. This result indicated that hands-on experience was more conducive to students with low academic level in geometry problem-solving.

In line with these findings, this would open the way for classroom practices. We can provide concrete manipulatives and meaningful operations in mathematics classroom, especially when students with lower academic level start to learn geometric knowledge. However, this is not to say that geometry reasoning only involves perception, but to recognize that sensorimotor information with the physical world is an integral part of learning, which has been neglected (Giardino, 2018).

## Limitations and future directions

The present study is a preliminary attempt to investigate the effect and the neural basis underlying the value of hands-on experience in geometry learning in middle school. We tried to bring educational neuroscience methods to geometry learning, and bridged the gap between neuroscience and mathematics education (Ansari and Lyons, 2016). Focusing on our experiences with NIRS in the school setting, chances and limitations exist in the current study should be noted. First, fNIRS has the potential to be used in the natural environment (Soltanlou et al., 2018), thus future research can be extended to real classrooms. Second, the small sample size and recruitment from one middle school may limit external validity. Future studies with more participants should be conducted. Finally, this study discusses the influence of hands-on experience on geometry learning. Subsequent studies can extend learning contents to algebraic problem solving.

## Conclusion

In summary, our findings indicated that hands-on experience improved geometry problems-solving performance for middle-school students. Hands-on operation caused the activation of the primary motor cortex and somatosensory association cortex during the learning process. Somatosensory association cortex was reactivated again during problem solving, which could effectively support geometry reasoning. Students with low academic level were more dependent on sensorimotor experience generated by perceptual and interactive richness of manipulatives.

## Data availability statement

The raw data supporting the conclusions of this article will be made available by the authors, without undue reservation.

## Ethics statement

The studies involving human participants were reviewed and approved by Nantong University. Written informed consent to participate in this study was provided by the participants’ legal guardian/next of kin. Written informed consent was obtained from the individual(s) for the publication of any identifiable images or data included in this article.

## Author contributions

LS, LD, and WZ co-designed and co-performed the experiments. LS conducted statistical analyses and wrote the manuscript. LD and DT examined the data and revised the manuscript. All authors contributed to the manuscript and approved the submitted version.

## Footnotes

1
http://www.pstnet.com/eprime.cfm
